# Cell–cell communications shape tumor microenvironment and predict clinical outcomes in clear cell renal carcinoma

**DOI:** 10.1186/s12967-022-03858-x

**Published:** 2023-02-10

**Authors:** Liu-xun Chen, Shen-jie Zeng, Xv-dong Liu, Hai-bin Tang, Jia-wu Wang, Qing Jiang

**Affiliations:** 1 Department of Urology, The Second Affiliated Hospital of Chongqing Medical University, No1. Yixueyuan Rd, Yuzhong District, Chongqing, China; 2 First Clinical Institution, Chongqing Medical University, Chongqing, China

**Keywords:** Cell–cell communications, Tumor microenvironment, Clear cell renal carcinoma, Prognosis, Immunotherapy

## Abstract

**Background:**

Cell–cell communications of various cell populations within tumor microenvironment play an essential role in primary tumor growth, metastasis evolution, and immune escape. Nevertheless, comprehensive investigation of cell–cell communications in the ccRCC (Clear cell renal carcinoma) microenvironment and how this interplay affects prognosis still remains limited.

**Methods:**

Intercellular communications were characterized by single-cell data. Firstly, we employed “CellChat” package to characterize intercellular communications across all types of cells in microenvironment in VHL mutated and non-mutated samples from 8 patients, respectively. And pseudotime trajectory analyses were performed with monocle analyses. Finally clinical prognosis and immunotherapy efficacy with different landscapes of intercellular interplay are evaluated by TCGA-KIRC and immunotherapy cohort.

**Results:**

Firstly, the VHL phenotype may be related to the intercellular communication landscape. And trajectory analysis reveals the potential relationship of cell–cell communication molecules with T cells and Myeloid cells differentiation. Furthermore, those molecules also correlate with the infiltration of T cells and Myeloid cells. A tumor cluster with highly expressed ligands was defined by quantitative analysis and transcription factor enrichment analysis, which was identified to be pivotal for intercellular communications in tumor microenvironment. Finally, bulk data indicates bulk that different clusters with different intercellular communications have significant predictive value for prognosis and distinguished immunotherapy efficiency.

**Conclusions:**

The intercellular communication landscapes of VHL wild and VHL mutant ccRCC vary. Intercellular communications within the tumor microenvironment also influence T cell and myeloid cell development and infiltration, as well as predict clinical prognosis and immunotherapy efficacy in ccRCC.

**Supplementary Information:**

The online version contains supplementary material available at 10.1186/s12967-022-03858-x.

## Introduction

Kidney cancer is among the 10 most common cancers in both men and women, representing 3.7% of all new cancer cases [1], and accounting for > 400,000 new cancer diagnoses and > 175,000 deaths worldwide each year, with an increasing incidence [2]. Clear cell renal carcinoma (ccRCC) is the most common phenotype of kidney cancer, occurring in 70% to 75%, which is strongly associated with von Hippel-Lindau (VHL) gene mutation [3]. Systemic treatment of ccRCC has rapidly evolved in recent years, especially for patients with metastatic ccRCC. Although the 5-year survival rates have demonstrated some improvements, while the overall prognosis is still poor, limited by the complexity and heterogeneity of immunobiology of ccRCC [1, 4]. Driven by a series of genetic and epigenetic variation, tumor cells can acquire phenotypic heterogeneity that enable them to grow more aggressively, invade neighboring tissues, harvest treatment resistance, and metastasize to distant sites [5]. Therefore, dissecting the tumor heterogeneity can contribute to the effectiveness of oncology treatment.

Tumoral heterogeneity mainly arises through tumor microenvironment (TME) by triggering different selective pressure. The TME typically comprises immune cells, including T and B lymphocytes, tumor-associated macrophages (TAM), dendritic cells (DC), natural killer (NK) cells; stromal cells such as cancer-associated fibroblasts (CAFs), pericytes, and mesenchymal stromal cells [6]. These complex components in TME can interact and communicate with each other. The signaling events behind cell–cell communications (CCCs) are often mediated by interactions of various types of protein, encompassing ligand–receptor, receptor–receptor and extracellular matrix–receptor interactions [7]. The downstream transcriptomic programs of receiver cells are modulated, and thereby leading to alteration in biological behavior of tumor, such as tumorigenesis, tumor progression, therapy resistance, immune infiltration, and inflammation [8]. These CCCs can in turn further regulated distinct cell subpopulations infiltration and differentiation [9]. Furthermore, VHL gene, which is the most frequently mutated in ccRCC, was reported that its dysregulation altered the activities of receptor/ligand protein, such as chemokine receptor CXCR3, epidermal growth factor receptor (EGFR), and CD70 [10–12]. These studies revealed a broad and profound biological link between VHL gene and CCCs. Whereas comprehensive investigation of CCCs in the ccRCC microenvironment in the different contexts of VHL mutation or not are currently lacking. Besides, as an important immune regulator, how CCCs affects the statue of the immune microenvironment has not been revealed.

Maturation of single-cell technologies has opened a new frontier for profiling complexity of TME in high resolution, and also facilitates the further portrayal of intercellular interaction within TME after definition of cell identity. Furthermore, a portion of the bulk-seq integrates a wealth of clinical information, providing a landing point for single cell analysis to explore clinical significance. Thus, we have jointly analyzed the single-cell RNA seq and bulk RNA seq to elucidate the biological significance and potential clinical values of CCCs in the TME of ccRCC. Here, we compared the state of cellular communication in VHL mutated versus non-mutated ccRCC, and analyzed the biological significance of cellular communication in the differentiation of T cells and myeloid cells. Subsequently, we defined a subgroup of tumor cells with high expression levels of cell surface ligands and investigate its biological behaviors and potential implication in clinical practice.

## Methods and materials

### Data collection

Single-cell mRNA sequence (scRNA-seq) data from 4 ccRCC patients with 4 tumor samples (1 sample is assigned to 1 patient), 1 VHL-mutated sample and 3 VHL-wild samples, were collected from Young, et al. cohort [13] and another dataset from Long cohort [14] including 4 samples from 4 ccRCC patients, all the patients were VHL mutated in Long cohort. After preliminary sample integration and quality control of scRNA-seq data sets, we generated a gene expression matrix with 76,118 cells. Furthermore, we downloaded the TCGA genomic, transcriptomic, and clinical data used in this study are from TCGA-KIRC database (https://portal.gdc.cancer.gov/), containing 539 ccRCC samples. And we also obtained Braun, et al. cohort [15], consisting of advanced ccRCC patients receiving immunotherapy (n = 592). RNA-sequencing data (FPKM values) were transformed into transcripts per kilobase million (TPM) values with better comparability.

### Identification and visualization of cell types in ccRCC samples

We created Seurat objects in general and for individual cell types depending on the purpose of our studies by the Seurat package. The top 2000 most highly variable genes were extracted to conduct principal component analysis (PCA), and the top 20 principal components (PCs) were used for the next classification analysis. To assign markers for the cell types, we performed t-distributed stochastic neighbor embedding (t-SNE) dimensionality reduction algorithm to further summarize the distinct clusters in t-SNE plots. We employed annotated information supported by the previous research to determine the biological types of all cells and the marker genes for each cell type are shown in Additional file 1: Table S1. In addition, we applied “AddModuleScore” function to calculate scores of gene set in all cancer cells.

### Cell–cell communication analysis

We removed batch effect after integration of Young et al. cohort and Long cohort based on 5000 genes with the most significant variation using “IntegrateData” function. CellChat is a tool that can quantitatively infer and analyze intercellular communication networks from scRNA-seq data and contains ligand-receptor interaction databases (http://www.cellchat.org/). The interactions were identified and quantified based on the differentially over-expressed ligands and receptors for each cell group (p < 0.05). Function “netVisual_diffInteraction” was employed to depict the differences in strength of intercellular communication. Afterwards, to explain how multiple cell populations and signaling pathways coordinate and drive intercellular communication, non-negative matrix factorization (NMF) was performed to speculate the numbers of communication patterns by function “identifyCommunicationPatterns”. Finally, we extracted the major signaling inputs and outputs among all cell clusters and depicted them using scatter plots. By computing the Euclidean distance between any pair of the shared signaling pathways in the shared two-dimensional manifold scatter plot, we applied function “rankSimilarity” to identify the pathways with the most significant changes in VHL mutated and non-mutated samples. Lastly, we compared the communication probabilities of ligand-receptor pairs regulated by certain cell populations into other populations. This was done by setting the parameter compare in the function “netVisual_bubble”.

### Pseudotime trajectory analysis of CCCs molecules

To explore the relationship between pseudotime trajectories and CCCs molecules, we utilized Monocle package for single-cell pseudotime analysis under default parameters. Our criteria for selecting highly variable genes are as follows: 1. mean expression ≥ 0.1, 2. Dispersion _empirical ≥ 1 * dispersion _fit. Then we used the ‘plot_pseudotime_heatmap’ function to depict the dynamic expression of CCCs molecules in the process of cell differentiation.

### Transcription factor analysis

We applied SCENIC with the pySCENIC package (0.11.2) to investigate the dominant transcription factors in distinct tumor clusters in python. SCENIC reconstructs regulons (transcription factors and their target genes) accesses the activity of these regulons in individual cells and defines meaningful clusters of cells [16]. pySCENIC consists of two major processes. Establishment of co-expression network by GENIE3 and identification of targeted genes through motif-analysis by RcisTarget database. Matrix of tumor cells generated using Seurat was as input data. After running GENIE3, motif dataset (hg38_refseq-r80_10kb_up_and_down_tss.mc9nr. feather, hg38-tsscentered-10 kb 7species.mc9nr.feather) was used to construct regulons for each transcription factor.

### Functional enrichment analysis

After identifying the regulons for the transcription factors, we then used clusterProfler package to perform Gene Ontology (GO) enrichment analysis, cell components, and molecular function pathways. In addition, P-value < 0.05 and adjusted P-value (Q value) < 0.05 were considered statistically significant. The Kyoto Encyclopedia of Genes and Genomes (KEGG) pathway in the C2 category from the molecular signature database (MSigDB) was extracted for gene set variation analysis (GSVA) to speculate the enrichment scores for the samples.

### Inference of copy number for tumor cells

Copy number analysis was performed with the inferCNV (v1.8.1) package under default parameters. Furthermore, mast cells were selected as normal reference. The tumor cells were clustered with the Seurat package (resolution = 0.9), and the expression matrix of tumor cells were then as input for the analysis.

### CIBERSORTx and CIBERSORT analysis

Abundance of 22 immune cell types in the 539 primary KIRC samples and advanced ccRCC patients receiving immunotherapy (n = 592) were calculated using CIBERSORT and the LM22 signature matrix [17], and the algorithm was run for 100 permutations. Additionally, we calculate the relative abundance of cell types identified by scRNA-seq data in bulk RNA data using CIBERSORTx website (https://cibersortx.stanford.edu).

### Estimation of TME in TCGA cohort

Estimation of STromal and Immune cells in Malignant Tumors using Expression data (ESTIMATE) is a computerized algorithm to infer stromal and immune cells infiltration levels in tumor tissues based on bulk RNA-seq data. We adopted ESTIMATE algorithm to compute the immune and stromal score for TCGA-KIRC patients, all the parameters were set as default.

### Statistical analysis

Statistical analyses of scRNA-seq data were performed by Seurat, CellChat, Monocle, SCENIC, inferCNV, CIBERSORTx online tool and GSVA. Difference analysis was performed by Student’s t-test and Wilcoxon rank sum test, respectively. Pearson analysis was adopted to calculate the correlation of CCCs molecules and relative abundance of 22 types of immune cell. All statistical analyses were undergone on the R (version 4.0.3) software, except for the SCENIC analysis. And p-value below 0.05 was considered statistically significant in this research.

## Results

### Classification and definition of cell types based on scRNA-seq of ccRCC samples

**Fig. 1:**
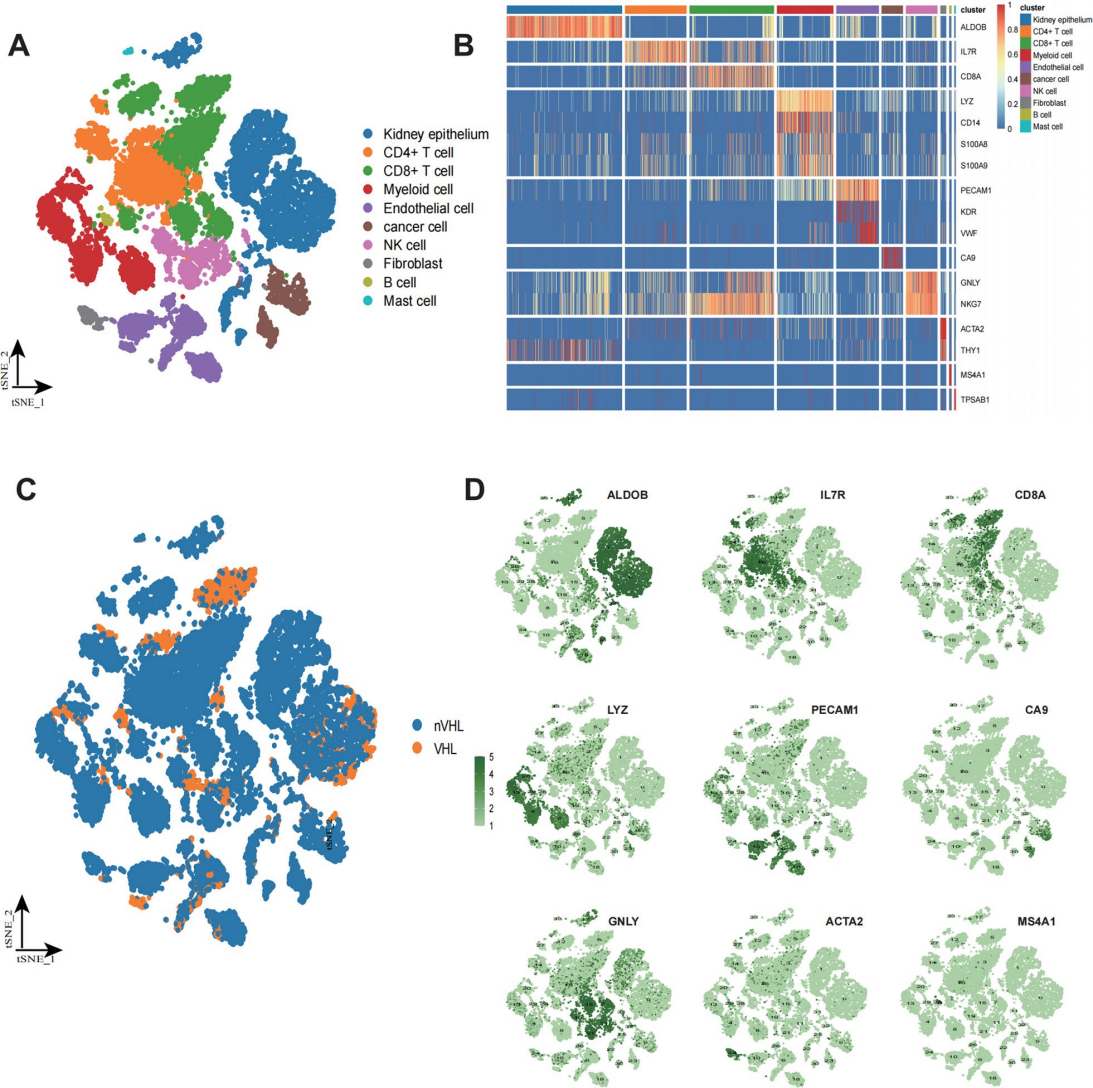
Classification and definition of cell types based on scRNA-seq of ccRCC samples. **A** Seurat t-distributed stochastic neighbor embedding (t-SNE) depiction of transcriptionally different cell populations in the tumor microenvironment from 4 patients. All cells are colored by their cellular annotations. **B** The heatmap displaying the distribution of cell type-specific genes in our samples. **C** tSNE view of all cells, color-marked by VHL mutation status. **D** The t-SNE plot shows the expression levels of cell type-specific genes across 10 clusters

We collected scRNA-seq dataset of *Young cohort* consisted of 1 VHL-mutated and 3 VHL-wild ccRCC patients to dissect the landscape of TME. We obtained 21,790 cells in total, and then performed t-SNE clustering. These cells were subsequently clustered into 10 cell subgroups, including kidney epithelium, CD4 + T cell, CD8 + T cell, myeloid cell, endothelial cell, cancer cell, NK cell, fibroblast, B cell, and mast cell (Fig. 1A) by identified the marker genes (Fig. 1B; Additional file 1: Table S1). we identified numerous CD4 + (expressing IL7R), and CD8 + T cell populations (expressing CD8A), natural killer (NK) populations (expressing GNLY, NKG7), small populations of B cells (expressing MS4A1/CD20) and population of myeloid (LYZ, CD14, S100A8, S100A9), Among non-immune clusters, we identified tumor cells (expressing CA9) and normal kidney epithelial cells (expressing ALDOB), and fibroblast (expressing ACTA2, THY1). In addition, visualization of the distribution of cells from VHL-mutated sample was conducted (Fig. 1C), and t-SNE plot displays the highly expressed marker genes in corresponding cell subgroups (Fig. 1D).

### Different landscapes of cell–cell communications in VHL-wild and VHL-mutant samples

**Fig. 2:**
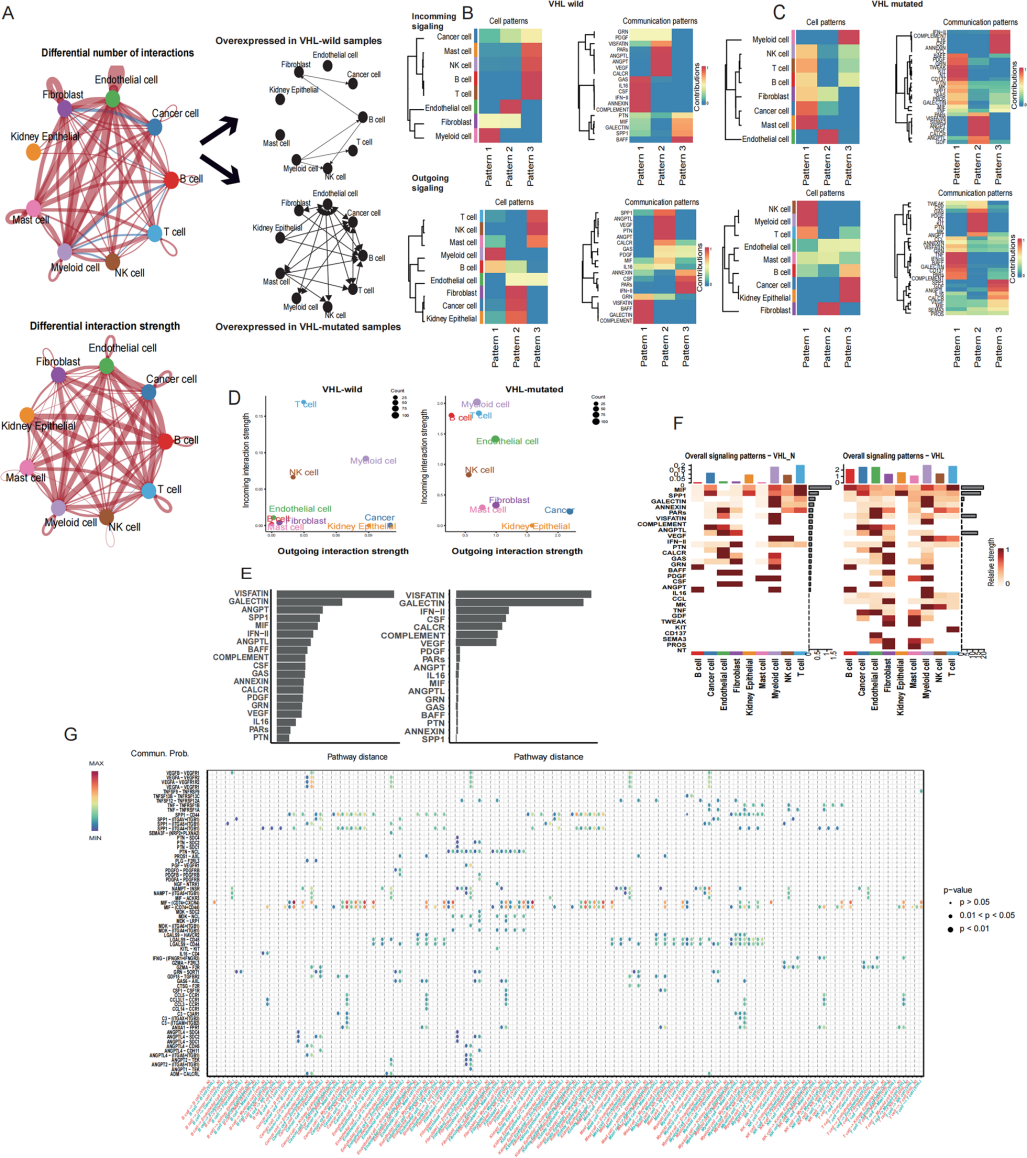
Cell–cell communications (CCCs) referenced by CellChat demonstrated notable alterations in receptors-ligands-mediated communications between VHL-wild and VHL-mutated cells in ccRCC. **A** Circle plots depicting the interaction numbers and interaction strength between VHL-wild and VHL-mutated cells. Blue lines indicate that the displayed communication is decreased in VHL-mutated cells, while red lines indicate that communication is increased in VHL-mutated cells compared with non-VHL group. The arrows indicate the direction of intercellular communication, which were also marked on the right in black for annotation. **B** NMF clusters based on the communication patterns of different cell components and types of ligands/receptors in VHL-wild group and **C** VHL-mutated group, The closer the color to red indicates the more contribution of the cell group or signaling pathway to each latent pattern. **D** scatter plot showing the intensity of the outgoing and incoming interactions in two-dimensional manifold. The size of the circles suggests the numbers of significantly expressed receptor-ligand pathways of different cell populations. **E** Based on the pairwise Euclidean distance in the shared two-dimensional manifold, we ranked the intersecting signaling pathways of VHL-wild and VHL-mutated groups. The length of the grey rectangle presents a difference in this pathway between the VHL-mutated and VHL-wild groups **F** The vertical axis is the cell that sends or receives the signal, and the horizontal axis is the pathway that receives or sends the signal. The color of the heat map represents the strength of the signal. The pillars on the upper and right sides are the accumulation of the strength of the vertical axis and the horizontal axis. **G** Comparison of Integral signal with superimposed input and output signals between VHL-wild and VHL-mutated groups, dot color reflects communication probabilities and dot size represents computed p-values computed from one-sided permutation test. Empty space means the communication probability is zero

Previous studies revealed that VHL-HIF signal plays an important role in immune cell differentiation [18]. To address whether this biological phenomenon caused by different mode of CCCs due to VHL variation, we applied CellChat to investigate the characteristics of CCCs in these two states. Due to the small number of samples with VHL mutations in the *M. D. Young cohort*, we integrated this cohort with *Z. Long cohort* via the “Seurat” package (Additional file 8: Figure S1A, B) Using known lineage markers, we identified kidney epithelium, T cell, myeloid cell, endothelial cell, cancer cell, NK cell, fibroblast, B cell, and mast cell. By comparing VHL mutant samples with wild samples using composition plot (Additional file 8: Figure S1C, D), we found the shifts in the percentage of cell populations. We analyzed interaction numbers and interaction strength in different types of cell subpopulations (Additional file 8: Figure S2A, B; Additional file 2: Table S2), then compared interaction numbers and strength in VHL wild and mutant cells on the overall level (Fig. 2A; Additional file 8: Figure S1C), which suggested wide range of variations of CCCs in the global communication pattern.

Additionally, to further explore the basis of the pathways mediating these changes, the signaling pathways associated with CCCs networks from VHL wild and mutated samples were obtained and mapped onto a shared two dimensions manifold and clustered into groups based on functional and topological similarity (Additional file 8: Figure S2D, E). Furthermore, how multiple types of cell subgroups coordinates to function could lead to alteration of CCCs (Fig. 2B, C). And we defined three patterns for outgoing and incoming signals of non-VHL and VHL mutated groups respectively based on non-negative matrix factorization to identify the collaborative mode of CCCs (Fig. 2B, C; Additional file 8: Figure S2F, G), and the results revealed that CCCs patterns of these two different groups shared the distinct cell subpopulation constitution, incoming and outgoing signaling activations. For example, pattern 3 in VHL-wild samples is comprised of Cancer cell, Mast cell, NK cell, B cell, and T cell in the levels of types of cells, while in VHL-mutated samples, pattern 3 is constituted of Myeloid cell, NK cell, T cell, B cell. These finding potentially reveals the different mode of collaboration of various cell types in different genomic background.

Further we explored the cell types that lead to changes in CCCs patterns, according to the incoming and outgoing interaction strength, we identified the sending and receiving signal cell populations with significant changes between these two groups, especially myeloid cell, B cell, endothelial cell, and fibroblast (Fig. 2D).

In addition to the alterations in CCCs activity in non-VHL and VHL mutated groups, ligands-receptors pathways play as an important role in both mediating and altering the CCCs landscape, by calculating the Euclidean distance between any pair of the shared signaling pathways in the two groups. We found the signaling pathways like Nicotinamide Phosphoribosyltransferase-Insulin (VISFATIN), Angioprotein (ANGPT), Secreted Phosphoprotein 1 (SPP1), and Macrophage Migration Inhibitory Factor (MIF) pathways with large distance in biological functions, while VISFATIN, Galectin 9 (GALECTIN), Interferon-II (IFN-II) pathways were uncovered large distance in network structure (Fig. 2E, F). In addition, prominently changes in their information flow of pathways in non-VHL mutant group as compared with VHL mutant group with following conditions: (i) turn off (such as CCL, CD137, TNF), or (iv) decrease (such as IFN-II, COMPLEMENT). The information flow for a given signaling pathway is defined by the sum of communication probability among all pairs of cell groups in the inferred network (Additional file 8: Figure S2H) [19]. We plotted and compared the L-R pairs and the communication probabilities of each cell group pair between the two groups, to display an overall picture of pathway variability (Fig. 2G). Next, the superimposed effects of the outgoing and incoming signals were identified and compared in both groups (Fig. 2G; Additional file 8: Figure S2I, J). Lastly. We discovered the alteration of ligand-receptor pathways (e.g., VEGF, VISFATIN) pathways (Additional file 8: Figure S2K). VEGF pathway mediates intercellular communication between a wider range of cell types in VHL mutant samples, which is also present in VISFATIN.

### CCCs modulated differentiation and infiltration of mononuclear/macrophage system

**Fig. 3:**
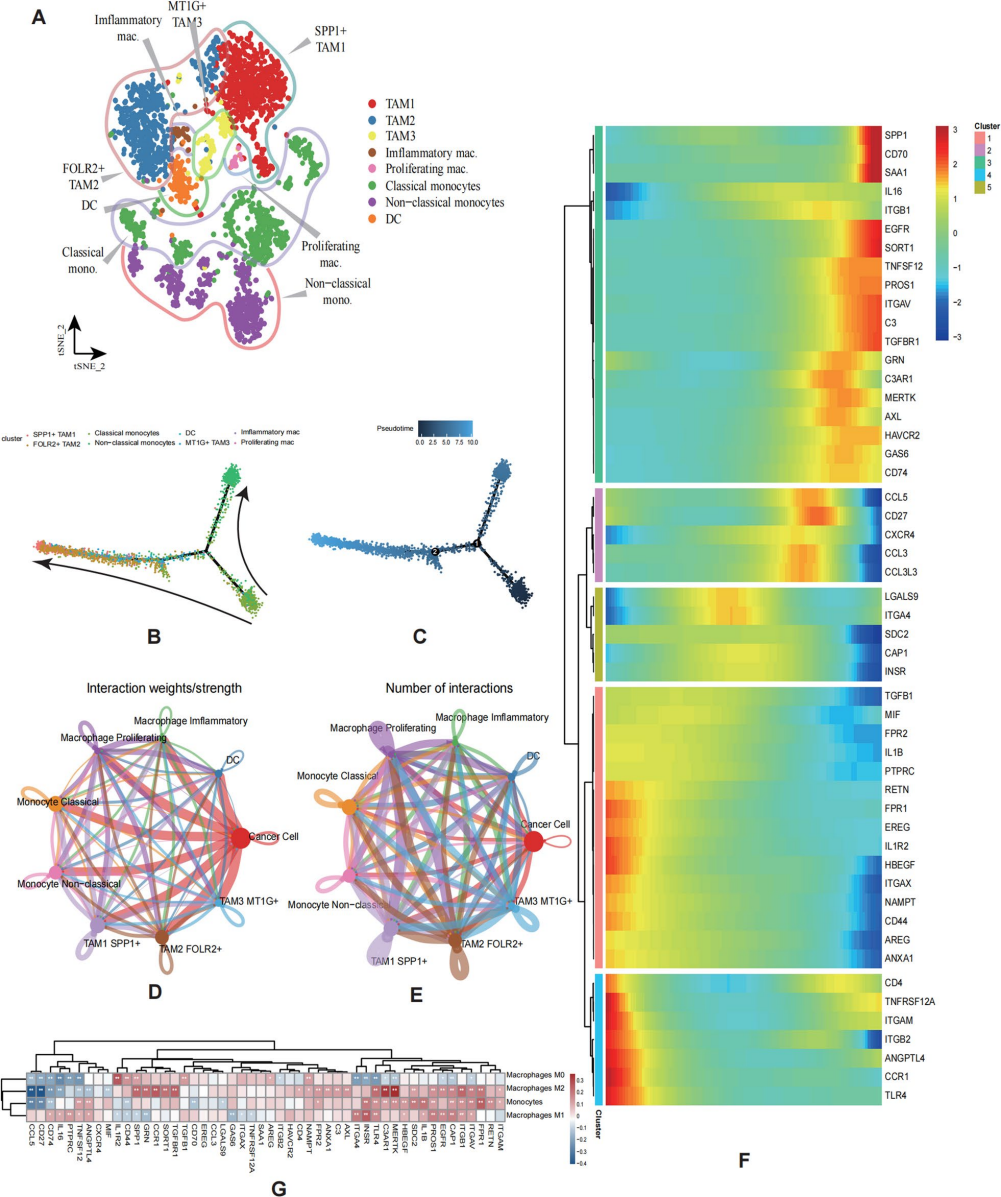
CCCs modulates the features of myeloid cells. **A** t-SNE plot depicting the myeloid cell subpopulations’ annotation. **B**,** C** Monocle analysis of myeloid cell subtypes, and the cells were ordered by pseudotime. **D**,** E** CCCs between main cell types of myeloid cells, tumor clusters by CellChat analysis based on interaction strength and numbers. **F** Trajectory Analysis suggests the role of CCCs molecules in process of myeloid differentiation. **G** Heatmap reveals the correlation between expression levels of CCCs molecules and relative abundance of immune cells, Welch’s t test: *P < 0.05; **P < 0.01; ***P < 0.001

CCCs coordinates the activities of multiple cell types required for immune response, therefore It is also important to investigate its effect on immunity to kidney cancer tumors. Mononuclear/macrophage reprogramming was identified to be strongly associated with tumor angiogenesis, immunosuppression, tissue remodeling and metastasis [20]. To further investigate the effects of tumor cell communication on the differentiation and infiltration of mononuclear/macrophage system, we extracted the myeloid cells in our data for classification and reannotation (Fig. 3A), which contains tumor associated macrophage1 (TAM1), TAM2, TAM3, inflammatory macrophage, proliferating macrophage, classic monocyte, and non-classic monocyte.

To address these questions, we used Monocle to perform differentiation trajectory analysis in mononuclear/macrophage system, based on the expression tendencies of FCGR3A (differentiation), S100A8 (stemness), S100A9 (stemness) (Additional file 8: Figure S3A). The results revealed that developmental hierarchy started with classical monocytes and non-classic monocytes and progressing towards SPP1 + TAM1 and FOLR2 + TAM2 (Fig. 3B, C). To confirm the CCCs-related molecules which probably modulate the process of mononuclear/macrophage system differentiation, we applied CellChat to visualize the communication strength, number and extract the key signaling pathways (Fig. 3D, E; Additional file 3: Table S3). We then analyzed changes in the expression of these receptor/ligand molecules during mononuclear/macrophage differentiation, and these molecules with remarkable variation were depicted on the heatmap (Fig. 3F), which indirectly suggested that these ligand/receptor molecules potentially influence the differentiation of mononuclear/macrophage system.

Subsequently, we confirmed these findings via bulk RNA-seq data. The mononuclear/macrophage system infiltration promotes cancer fibrosis and regulates the sensitivity of chemotherapy [21]. Applied to CIBERSORT revealed relative immune cell composition. Then, we used the “pearson” algorithm to calculate the correlation of M0, M1, M2 and monocytes with the expression of the screened receptor ligands (Fig. 3G). The analysis suggested the broad correlation between receptor/ligand gene expression and mononuclear/macrophage infiltration. Comprehensively, CCCs related molecules including C3, EGFR, HAVCR2, and so on, could influence the differentiation state and number of infiltrating myeloid in TME of ccRCC.

### Differentiation and infiltration of CD4/CD8 + T cell were influenced by CCCs

**Fig. 4:**
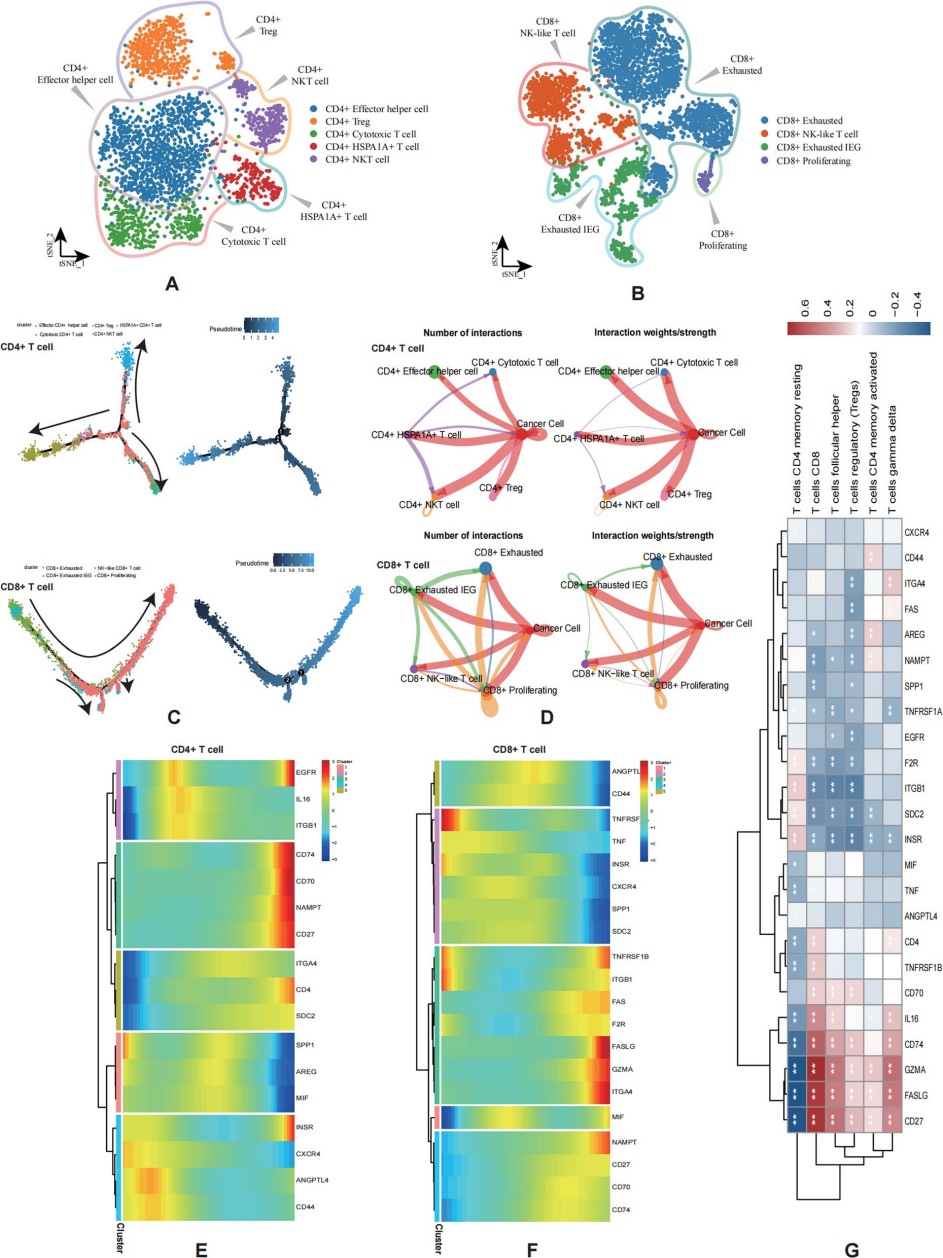
Differentiation and infiltration of T cells were influenced by CCCs. **A**,** B** Cell annotation of CD4 + T cells and CD8 + T cells by t-SNE plot, respectively. **C** Monocle analysis of CD4 + T cells and CD8 + T cells, which are ordered by pseudotime. **D** Network plot displaying the interaction strength and numbers of communication between CD4 + T cells, CD8 + T cells and tumor cells respectively. Welch’s t test: *P < 0.05; **P < 0.01; ***P < 0.001. **E**,** F** Expression conditions of CCCs molecules in the differentiation of CD4 + T cells and CD8 + T cells, respectively. **G** Heatmap demonstrating that CCCs molecules correlate with T cell infiltration in tumors

To confirm whether the similar biological efficacy mediated by CCCs occurs on T cell, we then clustered all the CD4 + T cells into CD4 + effector helper cell, CD4 + Treg, CD4 + cytotoxic T cell, CD4 + HSPA1A + T cell, and CD4 + NKT cell. And CD8 + T cells were clustered into CD8 + exhausted, CD8 + NK-like T cell, CD8 + exhausted immediate-early genes (IEG), and CD8 + Proliferating, repectively (Fig. 4A, B). We identified direction of differentiation of CD4 + T cells according to the expression shift of GZMK (activated), GZMA (activated), and CD27 (exhausted) (Additional file 8: Figure S3B), and TBX21 (stemness), TCF7 (stemness), and TOX (differentiation) expression levels were used to determine the differentiation direction of CD8 + T cells (Additional file 8: Figure S3C). Based on differentiation trajectory analysis, we demonstrated the divergent trajectory from HSPA1A + CD4 + T cell, cytotoxic CD4 + T cell to CD4 + Treg, and also defined the differentiation trajectory from CD8 + exhausted IEG to CD8 + exhausted (Fig. 4C). We subsequently conducted the visualization of the communication landscape between CD4 + and CD8 T + cells and tumor cells, respectively (Fig. 4D; Additional file 3: Table S3), and extracted the core receptor-ligand pathway for further analysis. We then respectively showed the expression trajectories of these ligands/receptors on CD4 + /CD8 + T cell differentiation using heatmaps (Fig. 4E, F). Correlation analysis revealed the wild associations between various types of T cell and CCCs related molecules (Fig. 4G). Among them, GZMA, FASLG, and CD27 were found to be most positively correlated with infiltration of CD8 + T cell, and negatively correlated with T cells CD4 memory resting. In general, the above results illustrated the profound significance of CCCs in T cell differentiation and infiltration.

### Identification of a tumor cluster with high ligand expression

**Fig. 5:**
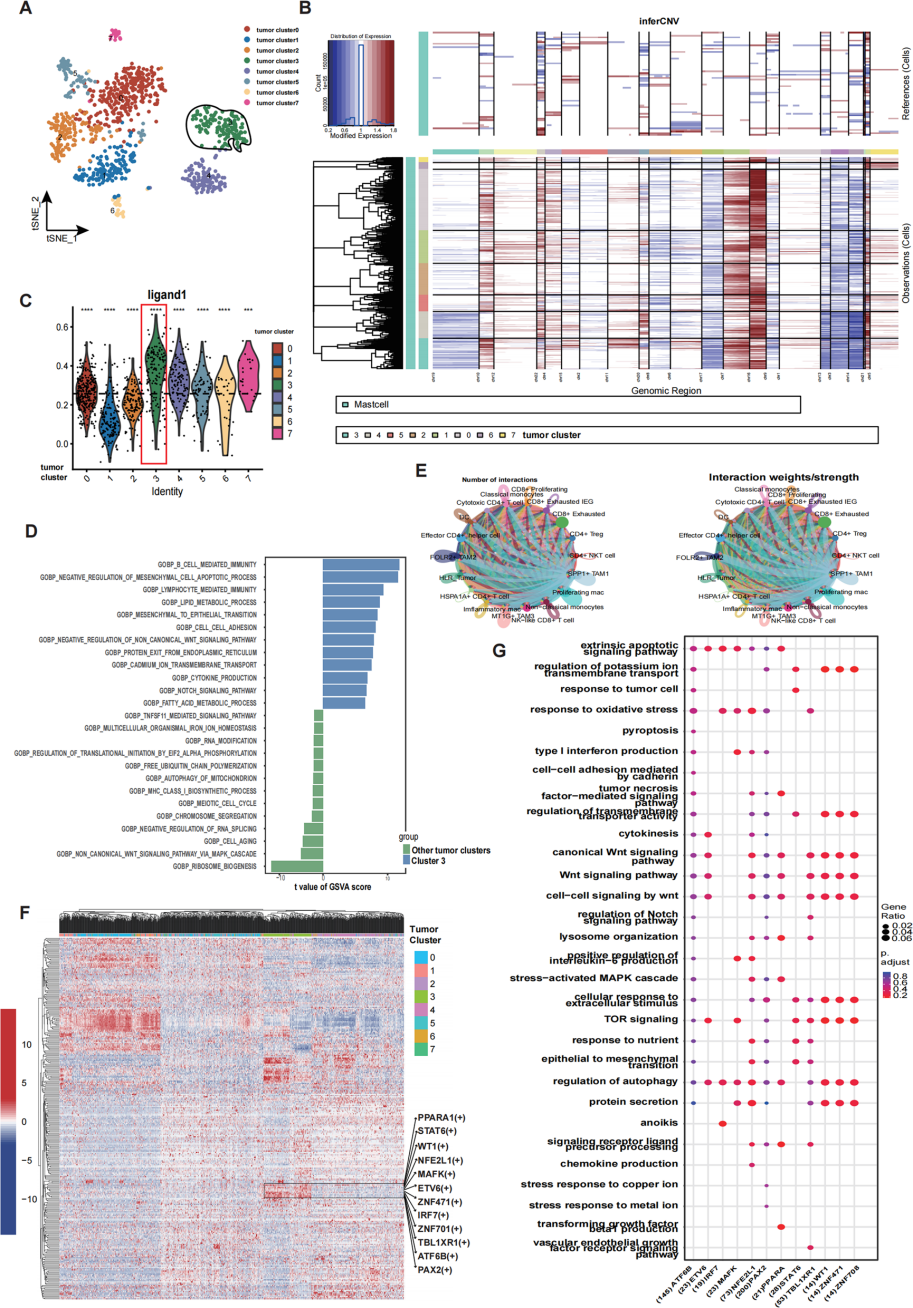
Identification of tumor cluster with high expression of ligands. **A** t-SNE plot displaying classification of tumor cells. **B** Inferred copy number variations of tumor cells were used to estimate the robustness of classifications. Blue indicates low modified expression, inferring to genomic loss; red indicates high modified gene expression, inferring genomic gain. Internal reference cells refer to mast cells. Observations refer to putative malignant epithelial cells. Genomic regions (chromosomes) are labeled and color-coded. **C** Violin plot demonstrates the distribution of ligand expression among cancer cells, Welch’s t test: *P < 0.05; **P < 0.01; ***P < 0.001, ****P < 0.0001 **D** Gene set variation analysis (GSVA) showing the enriched pathways of tumor cluster 3 compared with other tumor clusters. **E** CCCs between tumor cluster 3 with myeloid cells and T Cells. The bars to the right suggest that the pathways are upregulated in tumor cluster3, with longer bars suggesting more significant variance values. **F** The expression programs of transcription factors are heterogenous among different tumor clusters, the colors indicate the AUCell regulon activity of the transcription factors (TFs) as red (highly active) and blue (lowly active). TFs upregulated in tumor cluster 3 are marked on the right. **G** GO analysis of highly expressed transcription factors of tumor cluster 3 targeting downstream genes. The vertical coordinate represents the enriched pathways, and the horizontal coordinate represents the genes regulated downstream of the transcription factor (the number is the number of genes) predicted from the cistarget database

Tumor cells are important components in TME and are inevitably influenced by small molecules, immune factors and receptor-ligand pathways in the surrounding environment, we therefore aimed to investigate the biological characteristics and potential clinical translational significance of this cluster of tumor cells. Following tumor cells classification (Fig. 5A), we determined 8 clusters by t-SNE algorithms. To investigate whether these clusters are genomic differences, inferCNV analysis was applied to reveal that the eight tumor clusters are characterized by different copy number variants (Fig. 5B). We further found that the malignant cells have more copy number expansion signatures and deletions compared to other mast cells. Furthermore, malignant cells in cluster 3 indicates that there are more copy number deletions in chromosome (chr)3, chr9, chr13, and chr14, while the copy number gains in chr6, chr10, and chr11 are higher than other malignant cell clusters. Subsequently, we aimed to confirm the tumor cluster that was vulnerable to their surrounding environment. The result revealed that the ligand scores was highest in the tumor cluster 3 (Fig. 5C). We performed GSVA analysis to investigate the biological functions of tumor cluster 3, which demonstrated significant activation of B cell mediated immunity, mesenchymal epithelial transition, cell–cell adhesion, protein exit from endoplasmic reticulum, etc. (Fig. 5D, Additional file 4: Table S4), furthermore, we calculated and compared the GSVA scores of the pathways from hallmarks gene set from Molecular Signature Database (Additional file 8: Figure S3D) and then we conducted the CellChat analysis to depict the strength of this cluster's communication with myeloid cells and T cells, the results illustrated that tumor cluster 3 has an extensive and active ligand receptor interactions with these immune cells (Fig. 5E; Additional file 5: Table S5).

To further reveal the important role of Cluster 3 for CCCs within TME of ccRCC and potential mechanisms of differentiation. We performed SCENIC (single-cell regulatory network inference and clustering) analysis to conjecture regulon activities, which utilizes both co-expression modules between transcription factors and candidate target genes, according to the databases of DNA binding motifs of transcription factors to infer significant gene regulation (Additional file 6: Table S6) by these selected transcription factors and [22], and a transcription factors together with its target genes comprise a regulon. Based on the activities of the regulons we determined, all the tumor cells in cluster 3 were assigned to the common regions in our heatmap (Fig. 5F), which indicated the robustness and stability of our clustering. We screened out the regulons in the boxed area, which are highly expressed in cluster 3. Then, we performed the GO analysis to infer the biological functions of the gene regulatory networks (Fig. 5G; Additional file 7: Table S7). GO analysis revealed that the regulon networks enriched in the pathways associated with membrane protein synthesis and localization, this further reveals why this tumor subpopulation is highly ligand-expressing and thus may play an important role in the regulation of the microenvironment.

### Immune microenvironmet heterogeneity induced by CCCs affacts clinical outcomes and immunotherapy efficacy

**Fig. 6:**
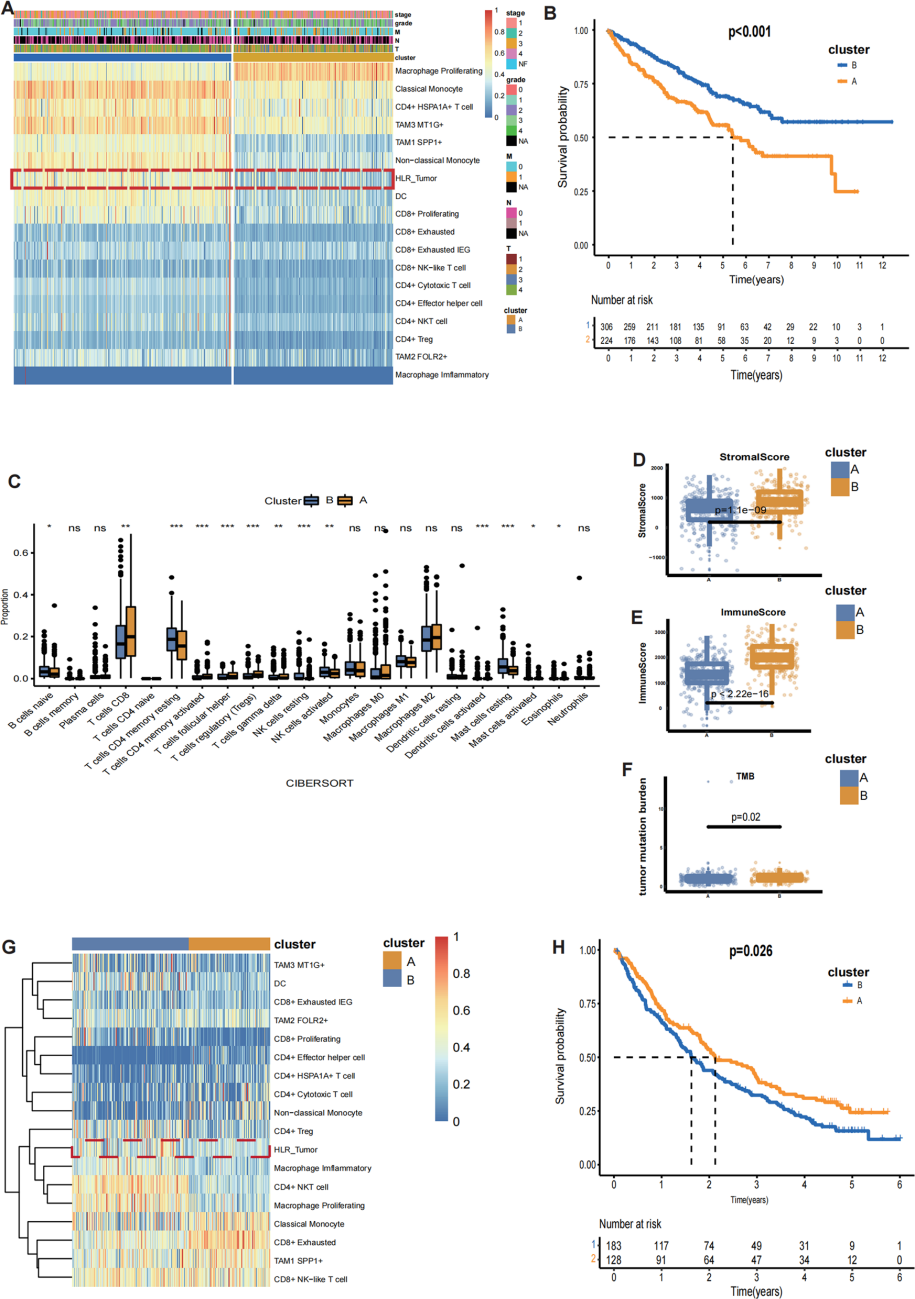
CCCs influences clinical prognosis and immunotherapy efficacy. **A** Unsupervised classification uncovers two clusters (Cluster A, Cluster B), depending on the fractions of myeloid cells, T cells and HLR-tumor cells calculated by CIBERSORTx of TCGA-KIRC cohort, the boxed section is the fraction of HLR tumor. **B** Kaplan–Meier plot demonstrates that ccRCC patients in Cluster B had a longer survival than Cluster A. **C** The fraction of immune cells is compared in Cluster A and Cluster B, *p < 0.05; **p < 0.01; ***p < 0.001; NS, not significant. **D**–**E** Box diagrams exhibited the correlation of stromal and immune calculated by ESTIMATE algorithm with clusters. **F** Patients in Cluster A have lower TMB levels than those in Cluster B. **G** Two clusters (Cluster 1 and Cluster 2) of David A. Braun, et al. cohort were identified by CIBERSORTx algorithm. **H** Kaplan–Meier curves of the OS for the Cluster A and Cluster B

Restricted by lack of clinical information on our single cell data and inclusion of patients, we have difficulty in determining the clinical translational value the high-ligand-expression tumor cluster. Therefore, we applied CIBERSORTx algorithm to calculate infiltration scores of cell subpopulations we identified in single-cell data to explore the clinical significance. Furthermore, we conducted unsupervised clustering to analyze ccRCC samples and classified patients into qualitatively different subgroups. Two distinct subgroups were ultimately identified, including 306 cases in Cluster A, and 224 cases in Cluster B (Fig. 6A). The heatmap demonstrated that the infiltration of HL tumor (High ligand tumor) is higher in Cluster B compared with Cluster A, and there is notable difference between these two cluster in Macrophage Proliferating, Classical Monocyte, CD4 + HSPA1A + T cell, TAM3 MT1G + , TAM1 SPP1 + , Non-classical Monocyte, HL_Tumor and dendritic cells. Furthermore, these clusters are predictive of disease outcome across TCGA (P < 0.001) cohorts (Fig. 6B).

CCCs as an important factor influencing the TME has profound implications for immunotherapy. The two clusters we obtained have different immune cell infiltration landscapes, also suggesting two different patterns of CCCs. Therefore, we inferred that there is distinct immunotherapy response between the Cluster A and Cluster B. To prove this inference, we then performed the following research. Firstly, we discovered that there was significantly different immune cell abundance in two clusters (Fig. 6C). Secondly, we applied the ESTIMATE algorithm to, to calculate the immune score and stromal score of each tumor tissue sample based on the gene expression matrix, and all parameters are set as default.

As expected, the immune parts and stromal parts were significantly upregulated in Cluster B (Fig. 6D, E). Thirdly, Genome analysis indicated that TMB is higher in Cluster B compared with Cluster A (p = 0.02, Fig. 6F), and waterfall plot demonstrated that the mutation rates of PBRM1 (45%) in Cluster B are significantly higher than these in Cluster A (33%), and BAP1 mutation rates were ultimately decreased in Cluster B (5%) compared with Cluster A (17%) (Additional file 8: Figure S4B, C). Lastly, we utilized David A. Braun, et al. cohort to further confirm our inference, this cohort contains information on patient survival and all patients received anti-PD-1 (Nivolumab) or anti-mTOR (Everolimus) therapy. Our classification result showed the heterogeneity in immune infiltration in both clusters as well (Fig. 6G), and the overall survival between these two clusters are also notably different (p = 0.026, Fig. 6H). In summary, different patterns of CCCs in ccRCC predicts different clinical outcomes and different immunotherapy response.

## Discussion

The effectiveness of tumor cell eliminating by immune cells is dominated by the intrinsic properties of these cell types and is closely associated with CCCs. Better understanding of how this interplay functions will contribute to explain how tumors could evade and become resistant to many forms of therapies at the cellular level [23]. However, comprehensive analysis of the CCCs in ccRCC is still lacking. We used single cell RNA sequencing to produce a single-cell transcriptomic atlas of CCCs in ccRCC, which allowed us to find unexpected biological features in various cell subpopulations. Joint analysis of bulk RNA-seq could provide us with a more comprehensive picture of the clinical translational values of these features.

In the beginning, to explore the relationship between VHL, the most commonly mutated gene in ccRCC, and CCCs, we used the “CellChat” package to illustrate the differences in the CCCs landscape on three levels. As a whole, we compared the overall intensity of CCCs among all cell subpopulations, and found the differences in the number of receptor-ligand pairs with or without VHL mutations. Further onwards, we applied the NMF algorithm to defined the distinct collaborative modes of CCCs in the two conditions, cell subpopulations that use the same or similar receptor-ligand pairs for cellular communication are designated as one NMF group and also considered to have the similar communication patterns. Finally, we analyzed the difference between the two conditions from the perspective of signaling pathways. Interestingly, we found that the CCCs activity of endothelial cells differed considerably between mutated and non-mutated cases of VHL, the results may be caused by activation of HIF mediated by VHL loss, which is implicated in angiogenesis [24]. These findings deepen our understanding of mutation-environment-induced tumorigenesis.

Subsequently, we analyzed the intercellular communication molecules with potential regulatory roles in the differentiation and infiltration of mononuclear macrophages and T cells, respectively. Monocle analysis revealed that the expressions of receptors and ligands changes as the immune cells differentiate, which indicated that CCCs could be closely associated with the differentiation of immune cells. Furthermore, the correlation analysis based on TCGA RNA bulk-seq, we found that CCCs influenced the infiltration of immune cells, which could further modulate the activities of anti-tumor immunity. Therefore, we speculated that tumor alters the immune cell infiltration and differentiation status via regulating the ligand/receptor pathways to benefit themselves. Moreover, the molecules we identified in the research may be potential clinical prognosis predictors and therapeutic targets.

Afterwards, we identified a tumor cluster with high expression of its ligand level, through analyzing the expression levels of ligands and performing GO analysis of TF downstream genes. This group of tumor cells may promote immune exhaustion, which lead to tumor immune escape. And the rich receptor-ligand interplay of this tumor group with T cells and myeloid cells was also uncovered, which further confirms that different cancer cell programs drive distinct immune interactions and differentiation status. Based on the some of the widely accepted predictors of immunotherapy and Braun, et al. cohort, highly-expressed ligands tumor subgroup suggests poor clinical outcomes in patients receiving immunotherapy, whereas result was reversed in treatment-free patients. This indicates a profound shift in the biological status of this tumor group as a result of the pharmacological effects of immunotherapy. And the opinion that immunotherapy induces reprogramming of CD8 cells and macrophages and remodeling of TME have also been confirmed by Kevin et al. [25].

In addition to VHL, there are many other mutations that affect immunotherapy efficacy, which brings challenges to our study. Although ccRCC is driven by a series of genetic mutation, dissecting the relationship between VHL and CCCs provides the basis for future works of the interplay between genetic background and the immune microenvironment. Furthermore, providing a complete single-cell profile of ccRCC patients can also be difficult due to lack of clinical samples, and bulk RNA-seq lacks precise cell annotations in tumor tissues. The lack of information on the actual spatial location leads us to speculate on the strength of cellular communication only through quantitative analysis, so we need to combine scRNA-seq data and spatial transcriptome data to elucidate the landscape of CCCs in the future.

Our study underscores that CCCs can be a key process in tumorigenesis, which probably drive the exhaustion of immune cells, and this regulatory process is inextricably linked to somatic mutations. We also identified numerous CCCs molecules, and the social internet they interact with orchestrates intrinsic properties of all cell subpopulations, which poses a path toward identification of novel therapeutic targets.

## Conclusion

In general, we analyzed the intercellular communications in ccRCC microenvironment via single-cell and bulk RNA sequencing. We explored the potential relationship between VHL, the most common mutation in kidney cancer, and intercellular communication, and then suggested that CCCs could alter the differentiation and infiltration of immune cells, which further influence clinical outcomes of ccRCC patients.

## Supplementary Information

**Additional file 1:** Markers of cell types used in this study.

**Additional file 2:** Cell-cell communication molecules among all cell types identified by CellChat algorithm.

**Additional file 3:** Cell-cell communication molecules among tumor cells and subgroups of myeloid cells, CD4+ T cells and CD8+ T cells identified by CellChat algorithm.

**Additional file 4:** List of GO pathways with differential activity in highly ligand-expressing tumor subgroups.

**Additional file 5:** Cell-cell communication molecules among HLR tumor cell cluster and subgroups of myeloid cells, CD4+ T cells and CD8+ T cells identified by CellChat algorithm.

**Additional file 6:** Identification of highly expressed transcription factors and downstream regulatory genes in different tumor subgroups based on SCENIC algorithm.

**Additional file 7:** GO enrichment results based on different transcription factors down-regulating gene sets, respectively.

**Additional file 8:** Document S1. Figures S1–S3.

## Data Availability

The data in this study can be downloaded from the Aditional materials provided by the article website (https://www.science.org/doi/pdf/10.1126/science.aat1699; https://www.ncbi.nlm.nih.gov/pmc/articles/PMC7499153/), which are all open access, the TCGA portal (https://portal.gdc.cancer.gov/) and the UCSC Xena (https://xenabrowser.net/datapages/).

## Abbreviations

ccRCC: Clear cell renal carcinoma
VHL: Von Hippel-Lindau
TME: Tumor microenvironment
TAM: Tumor-associated macrophages
DCs: Dendritic cells
NK: Natural killer
CAF: Cancer-associated fibroblasts
CCCs: Cell–cell communications
EGFR: Epidermal growth factor receptor
TCGA: The cancer genome atlas
KIRC: Kidney renal clear cell carcinoma
TPM: Transcripts per kilobase million
PCA: Principal component analysis
t-SNE: T-distributed stochastic neighbor embedding
NMF: Non-negative matrix factorization
GO: Gene Ontology
KEGG: Kyoto Encyclopedia of Genes and Genomes
MSigDB: Molecular signature database
GSVA: Gene set variation analysis
ESTIMATE: Estimation of STromal and Immune cells in Malignant Tumors using Expression data
HLR tumor: High ligand/receptor tumor
