# Supplementary material for: Cell–cell communications shape tumor microenvironment and predict clinical outcomes in clear cell renal carcinoma
Source: J Transl Med. 2023 Feb 10;21:113. doi: 10.1186/s12967-022-03858-x (PMC9921120; doi:10.1186/s12967-022-03858-x)
Supplement: Supplementary file 8 — Additional file 8: Document S1. Figures S1–S3. [file 12967_2022_3858_MOESM8_ESM.docx]

**Additional Figure**


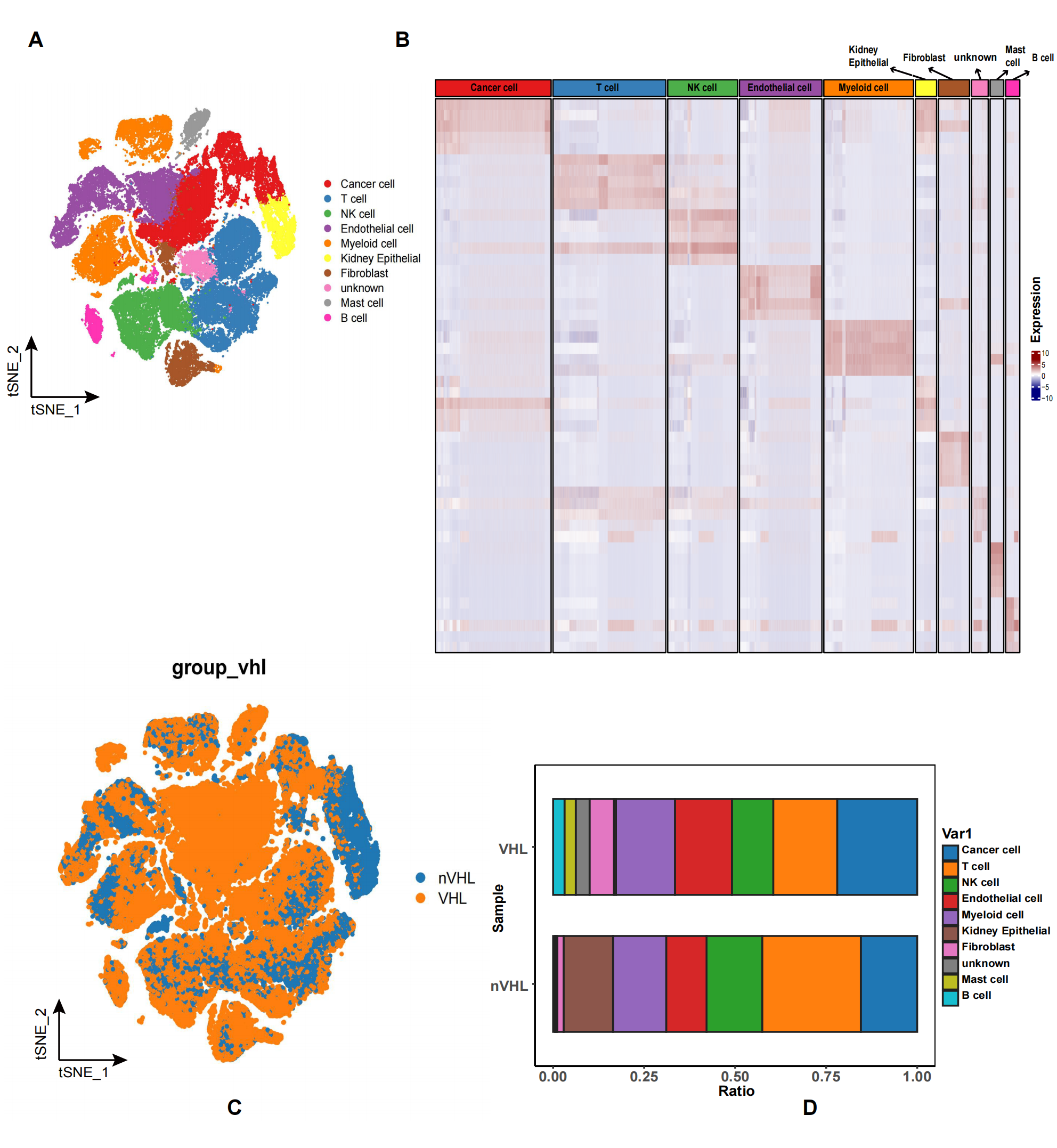


**Figure S1.** t-SNE representation for integrated from ***Z. Long cohort*** and ***M. D. Young cohort*** by CCA algorithm, color-coded for different cell types **(A)**, types of mutation (VHL-wild vs. VHL-mutated) **(B)**. Bar plots representing per cell type from left to right: the fraction of cell type in VHL mutated or non-mutated samples, respectively **(C)**. Heatmap of the top 10 marker genes for different cell types.


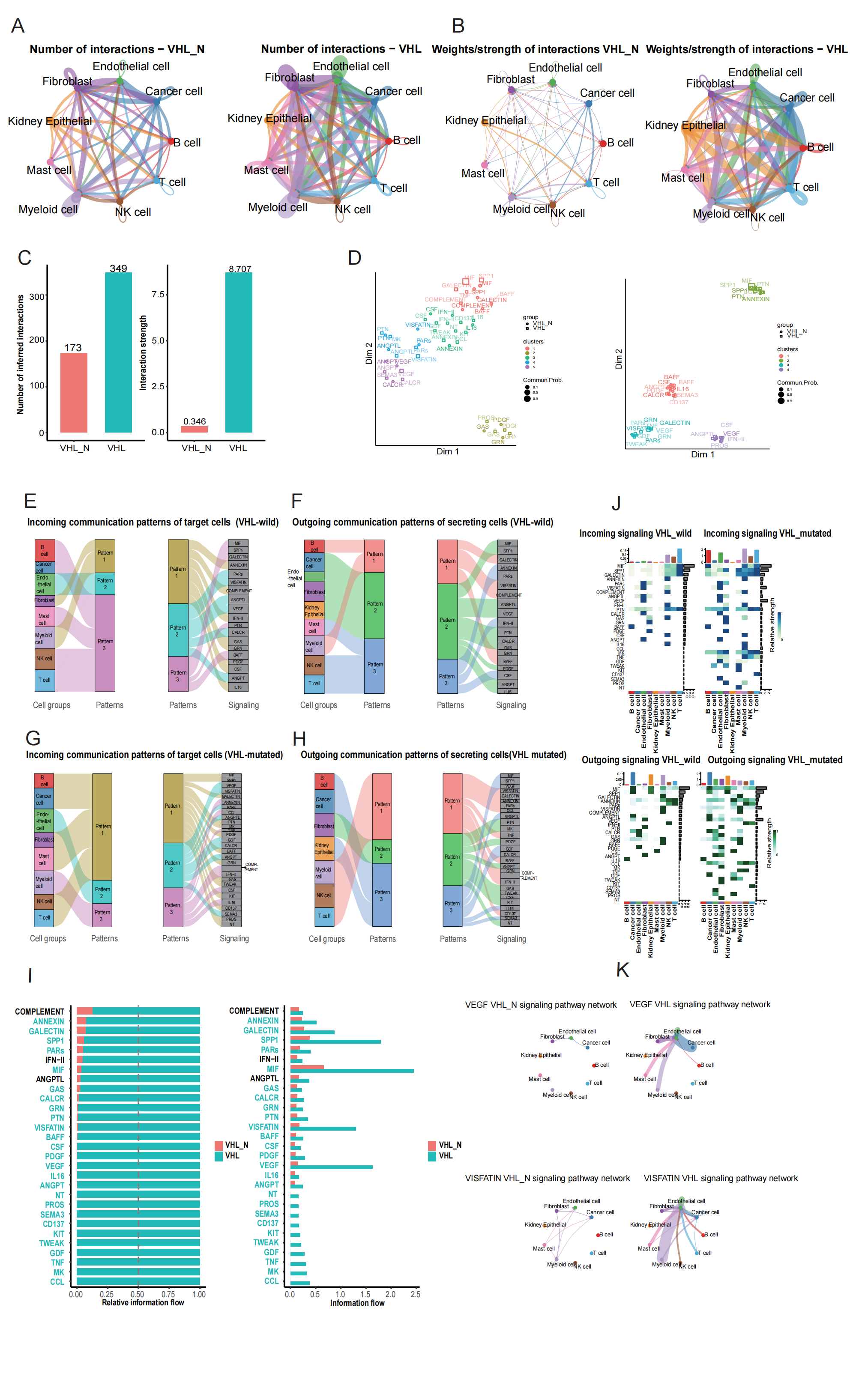


**Figure S2** CellChat analysis of VHL-wild and VHL-mutated renal cancer samples. Cell-cell communication of major cell types was analyzed separately in the VHL-wild ~~unmutated~~ **(A)** and mutated data **(B)**. The bar chart shows the difference in the number and intensity of cell-cell communications between the two data sets **(C)**. According to functional similarity **(D)** and structural similarity **(E)**, the signaling pathways are projected onto a two-dimensional manifold. Incoming and outgoing communications patterns calculated by NMF algorithm of VHL-wild **(F)** and VHL-mutated **(G)** data. All significant signaling pathways were ordered according to the differences in overall information flow in the network inferred between non-VHL mutated and VHL mutated **(H)**. Heatmap demonstrates the relative strength of incoming **(I)** and outgoing **(J)** signaling of VHL-wild and VHL-mutated data. **(K)** Network of inferred Vascular Endothelial Growth Factor (VEGF) and Nicotinamide Phosphoribosyltransferase-Insulin (VISFATIN) pathway between VHL-wild and VHL-mutated data.

**
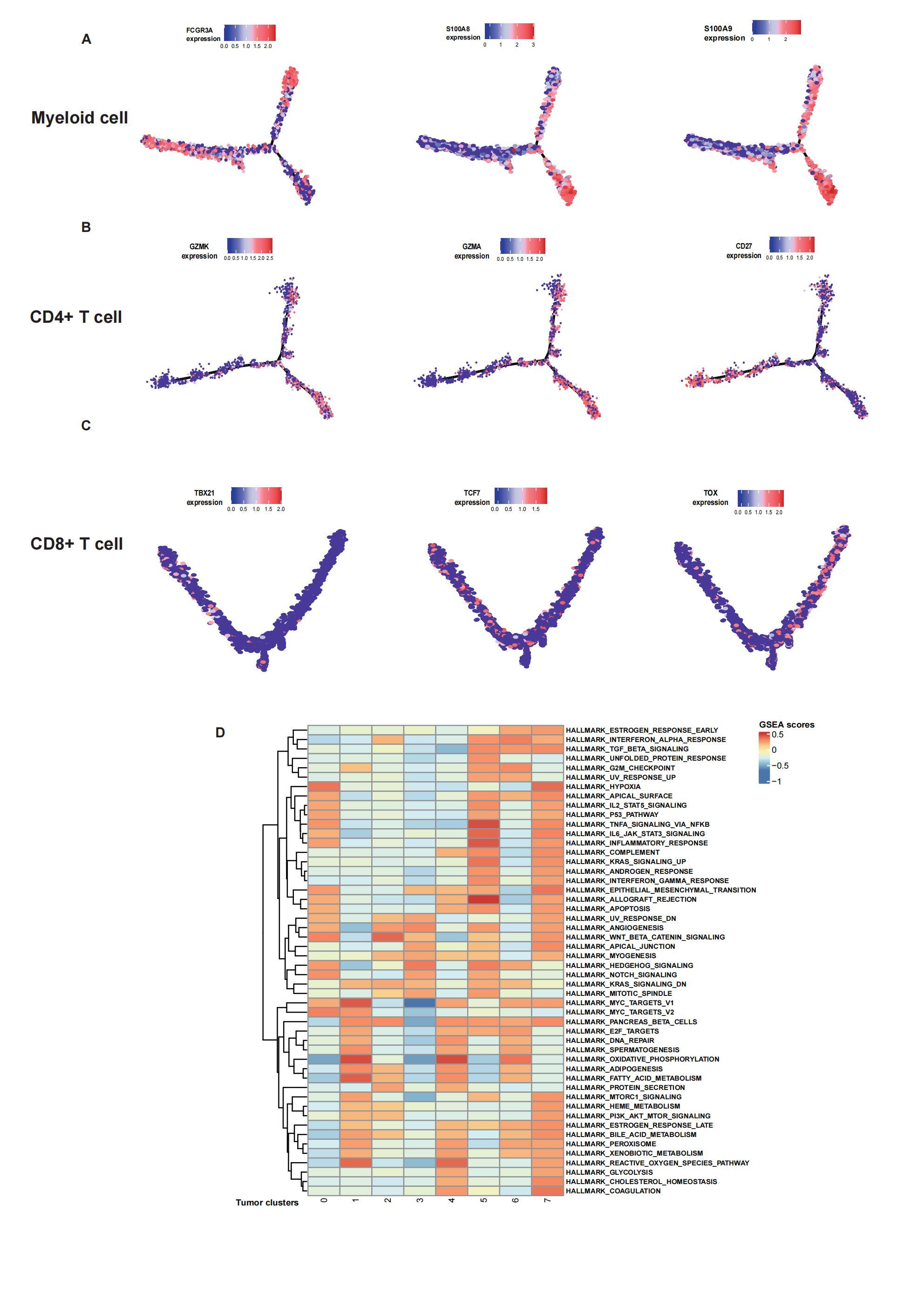
**

**Figure S3** The trajectory of differentiation predicted by monocle. **(A)** Identification of differentiation trajectory of myeloid cells based on the expression of FCGR3A, S100A8, and S100A9. **(B)** Differentiation trajectory of CD4+ T cells is identified according to the expression of GAMK, GAMZ, CD27. **(C)** The expression of TBX21, TCF7, and TOX indicates the differentiation trajectory of CD8+ T cells. **(D)** Heatmap showing core pathways activity from MsigDB database calculated by “GSVA package” for each tumor clusters.

**
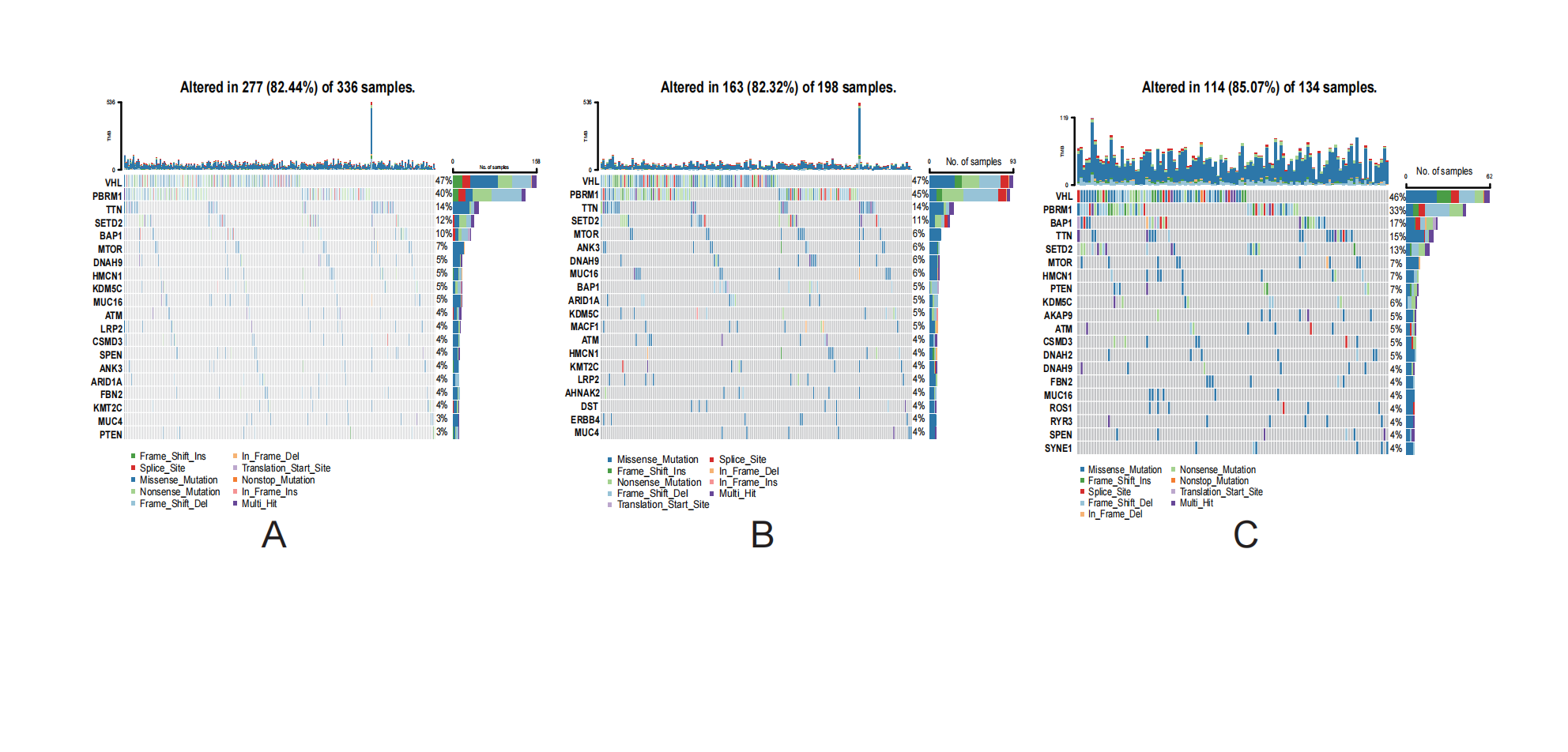
**

**Figure S4.** The oncoPrint was depicted based on TCGA somatic mutation data **(A)**, mutational landscape of high riskscore **(B)** and low riskscore groups **(C)**. Individual patients represented in each column. Single-nucleotide variants information in relation to colors is marked under the graph, The top bar plot indicates TMB per patient, whereas the right bar plot shows the mutation frequency of each gene in separate groups.
